# Effects of Alloy Composition, Hardness, and Milling Parameters on the Cutting Forces of Al-Li-Based Alloys

**DOI:** 10.3390/ma18122683

**Published:** 2025-06-06

**Authors:** Lida Radan, Victor Songmene, Agnes M. Samuel, Fawzy H. Samuel

**Affiliations:** Department of Mechanical Engineering, École de Technologie Supérieure, Montreal, QC H3C 1K3, Canada; lida.radan.1@ens.etsmtl.ca (L.R.); victor.songmene@etsmtl.ca (V.S.); agnesmsamuel@gmail.com (A.M.S.)

**Keywords:** aluminum–lithium alloys, machining, cutting force, heat treatment

## Abstract

This article explores how alloy composition, hardness, and machining parameters can affect the cutting forces encountered in aluminum–lithium (Al-Li)-based alloys. By analyzing Cu and Cu with Sc additions to Al-Li alloys and exposing them to various heat treatments to modify their hardness, this research was designed to evaluate milling performance under various feed rates, cutting speeds, and cooling conditions. The findings indicate that increased hardness leads to higher cutting forces, with Al-Li-Cu-Sc exhibiting the greatest resistance due to scandium’s grain-refining effect and the formation of stable precipitates. Statistical analyses identify the feed rate as the main parameter controlling cutting force, along with hardness and cooling conditions. Notably, wet machining consistently reduces cutting forces, especially in Al-Li-Cu-Sc alloys, enhancing machinability when using high cutting speeds. This work underscores the significance of selecting optimal machining parameters tailored to specific alloy compositions. These findings contribute to improved process efficiency, reduced tool wear, and enhanced productivity. Given the attractive characteristics of these alloys, i.e., their low weight and high strength, the insights from this study are particularly beneficial for aerospace applications where machining performance directly impacts component quality, cost, and overall operational efficiency.

## 1. Introduction

Aluminum–lithium (Al-Li) alloys have garnered significant attention in the aerospace and space industries due to their unique combination of lightweight properties, high strength, excellent fatigue resistance, and superior corrosion performance [1,2,3]. The addition of lithium, the lightest metallic element, significantly reduces the density of aluminum while forming δ′ (Al_3_Li) precipitates that enhance the mechanical properties of the alloy [4,5]. This reduction in density improves the specific stiffness and makes Al-Li alloys ideal for lightweight structural components in aerospace applications [6,7,8].

In addition to lithium, alloying elements like copper (Cu) and scandium (Sc) are often added to Al-Li alloys to further refine their microstructure, improve hardness, enhance thermal stability, and increase overall machinability. Al-Li alloy systems with Cu or Cu + Sc offer superior mechanical properties due to the formation of additional strengthening phases, such as Al_2_Cu (θ′) or Al_2_CuLi (T1), and coherent Al_3_Sc precipitates that enhance grain refinement and prevent recrystallization [9]. These advanced properties make Al-Li alloys increasingly attractive for high-performance applications, where both lightweight design and mechanical integrity are essential.

However, the machining of Al-Li alloys presents several challenges due to their relatively soft and ductile nature. These alloys are prone to machining-related issues such as built-up edge (BUE) formation, chip adhesion, surface smearing, and excessive tool wear. Such issues can compromise surface quality, dimensional accuracy, and tool life, making it essential to optimize machining parameters to improve the overall machinability of Al-Li-based components [10,11].

The cutting force is one of the key aspects of machining optimization; it provides critical insights into the forces exerted during material removal processes and plays a crucial role in determining tool wear, tool life, machining accuracy, and process stability. Excessive cutting forces can lead to tool deflection, chatter, vibration, and poor surface finish, all of which negatively affect machining outcomes [7,12]. By carefully selecting and optimizing cutting parameters (such as the feed rate, cutting speed, depth of cut, and cooling conditions), manufacturers can minimize cutting forces, improve tool longevity, enhance machining efficiency, and reduce production costs [13].

Various factors influence the cutting forces during the machining of Al-Li alloys, including material properties (such as hardness), lubrication, and heat treatment conditions. The microhardness of Al-Li alloys, which can vary depending on heat treatment and alloy composition, is a critical factor affecting cutting forces and overall machinability. Studies have shown that increasing alloy hardness generally increases cutting forces, although this can reduce BUE formation and improve tool performance in certain cases [14,15].

Mou et al. [15] highlighted the importance of surface integrity and demonstrated how feed rate, cutting speed, and lubrication conditions affect cutting forces and operational efficiency in milled Al-Li components. Wang [16] found that ultrasonic-assisted end milling can significantly reduce cutting forces and enhance both tool life and surface quality, particularly in high-speed machining operations.

Zedan et al. [14] studied the impact of copper (Cu) and magnesium (Mg) additions on the machinability of heat-treated Al-Si alloys and observed that the increased hardness due to Al2Cu phase formation led to higher cutting forces and rougher surface finishes. Similarly, Gonçalves et al. [9] demonstrated that precipitation hardening in Al-Si-Mg-Cu alloys enhances hardness but also increases cutting forces during machining. Marakini et al. [11] investigated the use of PVD-coated carbide tools for machining Al-Li alloys and found that optimized cutting speeds and feed rates can reduce cutting forces, minimize tool wear, and improve the overall machinability.

Despite these advancements, a comprehensive understanding of the cutting forces involved in machining Al-Li alloys with varying alloy compositions and heat treatment conditions remains elusive. This study was designed to fill this gap by conducting a detailed statistical analysis of the cutting forces generated during the milling of three specific Al-Li alloy systems: Al-Li and those containing Cu and Cu with Sc. This study investigated how significant process parameters (these being the alloy hardness, feed rate, cutting speed, and cooling mode) influenced cutting force trends. By systematically evaluating these factors, the research aimed to optimize the machinability of Al-Li alloys, contributing to more efficient manufacturing processes and the production of next-generation lightweight aerospace components.

## 2. Materials and Methods

**Figure 1:**
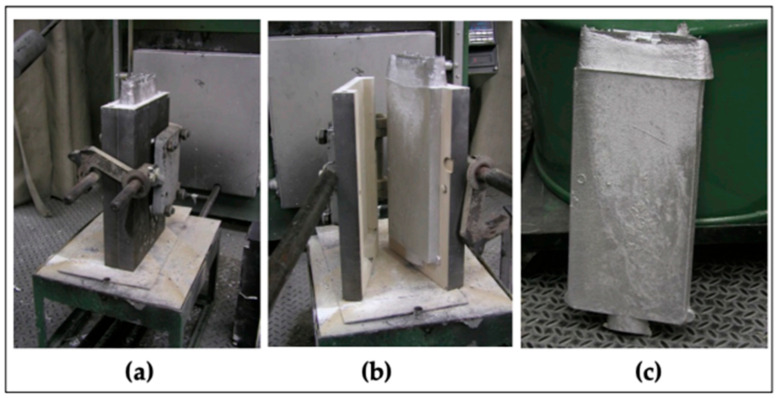
(**a**) The shape of the metallic mold used, (**b**) the casting inside the mold, (**c**) the final casting (103 mm in length, 41 mm in width, and 33 mm in thickness) [16].

Three aluminum–lithium (Al-Li) alloys—Al-Li, Al-Li-Cu, and Al-Li-Cu-Sc—were examined in this work. The Al-Li base alloy was Al-4 wt.%Li, to which alloying elements, including copper and scandium, were introduced in the form of pure Cu, Al-2 wt.%Sc master alloy, and commercially pure aluminum (99.5%). The method of casting the present alloys is depicted in Figure 1a using a metallic permanent mold preheated at about 450 °C (for more details see Ref. [16]). The final compositions of the alloys are presented in Table 1.

Various heat treatments were applied, and the corresponding effects on the microhardness and microstructural features of these alloys were studied, as reported in [16]. From each alloy, samples (10 mm × 15 mm × 20 mm) were cut from the casting and separated into three sets, keeping one set in the as-cast condition; another set was solution heat-treated and then quenched in warm water and kept at −10 °C until testing, while the third set was solution heat-treated, quenched in warm water, and then artificially aged at 130 °C (or 150 °C) for times ranging from 1 h to 45 h at the aging temperature. Details of the heat treatments used for the three alloys studied are specified in Table 2.

Heat treatment was applied to the alloys to assess the effect of thermal processing on their microstructural features and mechanical properties. For the Al-Li alloy, samples underwent solution heat treatment at 580 °C (a temperature above its solidus) for 1 h, followed by quenching in warm water, and were subsequently aged at 130 °C and 150 °C for durations ranging from 1 to 45 h. Due to the incipient melting associated with the presence of about 3% Cu [17] in the Al-Li-Cu and Al-Li-Cu-Sc alloys, samples were solution heat-treated at 505 °C for 5 h, quenched in warm water, and artificially aged at 160 °C, 180 °C, and 200 °C for durations between 5 and 30 h (Table 2).

A total of 58 samples were used. All heat treatments were carried out in a Thermolyne Electric Furnace (Thermo Fisher Scientific, Waltham, MA, USA). Samples were mounted in Bakelite and then polished to a fine finish following standard grinding and polishing procedures as described elsewhere [16].

Microhardness measurements for all samples were carried out using a load of 100 g (0.9807 N) applied during measurement. Ten indentations were made on each sample, and the average value was considered as representing the microhardness of the specified sample.

Figure 1 shows the development of hardness as a function of the natural aging time. The Al-Li alloy exhibits an increase in hardness with an escalation in aging time. Solution treatment for 1 h at 580 °C followed by artificial aging at 150 °C causes a change in hardness from 44.7 HV to its peak value of 97.2 HV after 45 h. The primary cause of the observed increase in hardness in the Al-Li alloy was the formation of the δ’ phase. The Al-Li-Cu and Al-Li-Cu-Sc alloys also show an increase in hardness with an escalation in aging time up to 180 °C, followed by a decrease due to the commencement of overaging, caused by the coarsening of the precipitated particles. For all heat treatments, the presence of Cu and Sc maximized the peak hardness. The strengthening effect of the solution and aging treatments was the main reason for the improvement in hardness. Artificial aging at 180 °C for 20 h provided peak hardness values of 163.6 HV and 182.6 HV for the Al-Li-Cu and Al-Li-Cu-Sc alloys, respectively.

In addition, the Al-Li-Cu-Sc alloy exhibited the highest hardness values. This was ascribed to the presence of Al-Sc precipitates, which, having a very small size (10–100 nm), create many obstructions for dislocations to pass through. The addition of Sc produces a high density of fine, uniformly distributed precipitates within the aluminum grains, contributing even more to this strengthening [6,17].

To identify the intermetallic phases present in the alloys studied, a scanning electron microscope (SEM-Hitachi High-Tech Corporation, Ibaraki, Japan) equipped with an energy-dispersive X-ray spectrometer (EDS- Madison, WI, USA) was employed, while a field emission scanning electron microscope (FESEM-Hitachi High-Tech Corporation, Ibaraki, Japan) was used to characterize the strengthening precipitates resulting from the various heat treatments and to examine the deep-etched solutionized samples to characterize the silicon particles. The SEM used attached to an EDAX Phoenix system designed for image acquisition and energy-dispersive X-ray (EDS) analysis. The SEM was operated at a voltage of 20 kV, with a maximum filament current of 3 amperes.

Table 3 outlines the experimental machining variables and their associated levels. Qualitative factors included the workpiece materials and cooling mode, while the other factors were treated as quantitative variables. The corresponding levels were selected based on industrial recommendations.

The milling operation was performed on a block with dimensions of 103 mm in length, 41 mm in width, and 33 mm in depth. The investigation encompassed 18 distinct conditions, derived from a Cartesian combination of three key factors: the alloy material, heat treatment variations, and the type of machining environment (dry or wet), yielding 18 unique combinations (3 × 3 × 2 = 18). Each condition was subjected to nine different tool paths, determined by adjusting the cutting speed and feed rate parameters, and required the carrying out of 162 experiments.

All 162 experiments were carried out under both dry and wet milling environments, employing a 3-axis CNC machine tool with specific attributes, including a 50 kW power output, 28,000 rpm rotational speed, and a torque of 50 Nm. Milling operations were performed utilizing non-coated carbide end mills featuring a three-flute design (z = 3) and 10 mm in diameter. To maintain consistency in test conditions, the tools were changed every nine tool paths, resulting in the use of eighteen unique tools during the experiments. In this study, the three cutting force components (Fx, Fy, and Fz) were measured using a three-axis dynamometer (Kistler, model 9255-B), which was mounted on the milling machine and connected to a data acquisition system for analysis. To obtain the cutting force signal, a sampling frequency of 12 kHz was employed.

## 3. Presentation and Discussion of Results

**Figure 2:**
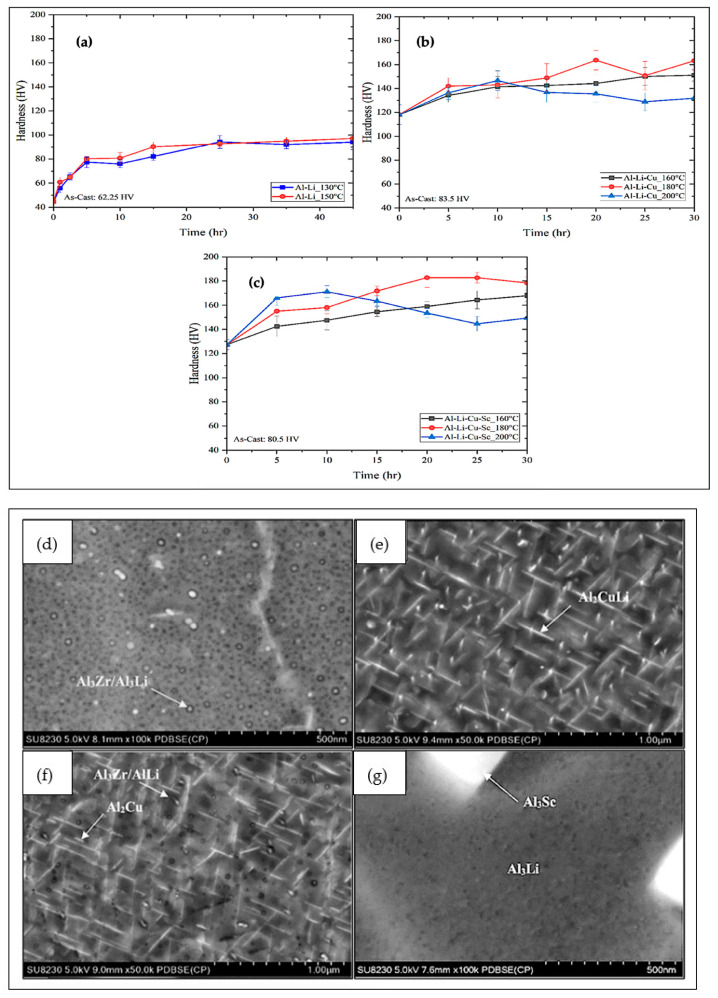
Variation in Vickers microhardness with increase in aging time in (**a**) Al-Li, (**b**) Al-Li-Cu, and (**c**) Al-Li-Cu-Sc alloy samples heat-treated at different temperatures [16]. SEM images obtained after aging treatment of the alloys studied: (**d**) Al-Li alloy, (**e**) Al-Li-Cu alloy, (**f**,**g**) Al-Li-Cu-Sc alloy.

Figure 2d–g displays various SEM images of the alloys studied. Figure 2d depicts a low-magnification image of the Al-Li alloy after aging, showing δ′ (Al_3_Li) precipitates that appear homogenously distributed throughout the α-Al matrix. The higher-magnification images presented in Figure 2e,f indicate that the precipitates change in shape when Cu and Sc are added to the Al-Li alloy. As Figure 2c reveals, small spherical particles are also visible in the microstructure of the Al-Li-Cu-Sc alloy. The larger plate-like precipitates form at the grain boundaries, while smaller ones occur within the grains. The presence of Sc in the alloy increases the density of precipitates and disperses them more uniformly within the matrix (Figure 2f). Also, it is well known that, in facilitating the formation of the Al_3_Sc phase, Sc significantly improves the mechanical properties of aluminum alloys (Figure 2g). The dispersed Al_3_Sc particles enhance the alloy strength, substantially reinforce the substructure, and hinder the process of recrystallization [6].

### 3.1. Statistical Analysis of Cutting Force in the Machining of Al-Li-Based Alloys

In the present study, the analysis of variance (ANOVA) was used to determine statistically significant cutting factors in the dependent machining variables studied. It involves studying the variability due to the difference in the effects of different factors and their levels. The significance of the factors was determined via the comparison of the F-ratio between them with a *p*-value below 0.05 (95% confidence level). Only significant factors and their interactions were considered in the analysis [18].

**Figure 3:**
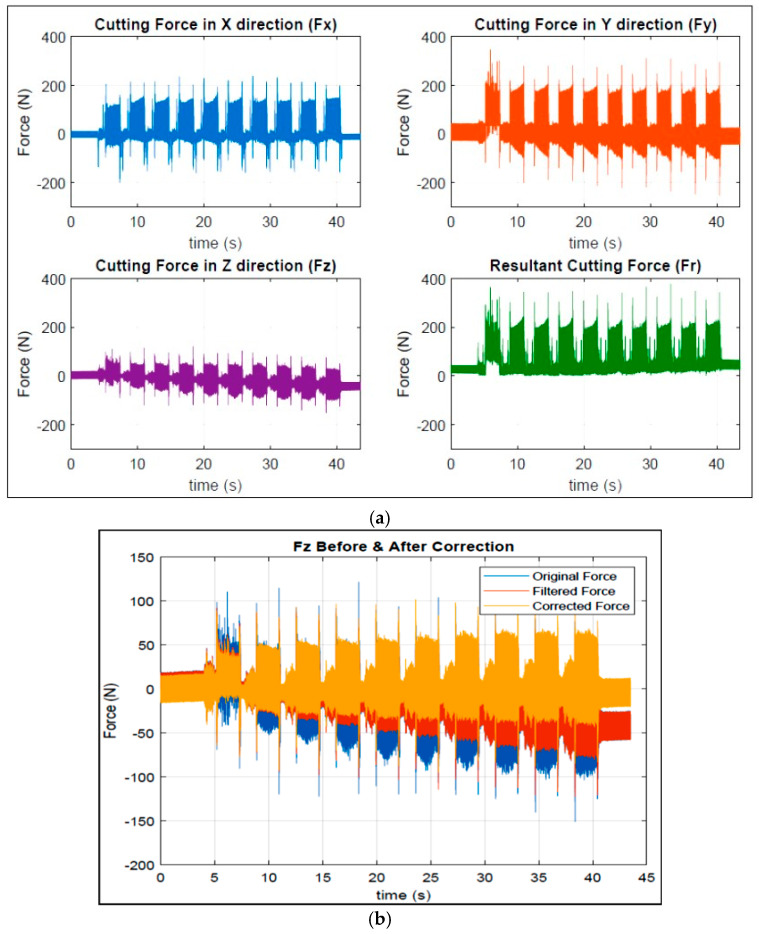
(**a**) Variation in cutting forces as a function of the cutting time: Fx, Fy, Fz, and Fr for the base alloy. (**b**) Cutting forces for the Fz component before and after correction.

To determine the cutting forces along the X-, Y-, and Z-axes, the raw data generated from the data acquisition system attached to the dynamometer mounted on the milling machine were transformed into frequencies by applying the Fourier transform method. Thereafter, filtration was used digitally to remove unnecessary frequencies (which are normally noises generated from the motor of the machine). Figure 3b represents the cycle correction process that was carried out. Data processing was accomplished by applying MATLAB software (Version 24.2), where the radial force Fr is defined as follows:

Fr = √(Fx^2^ + Fy^2^ + Fz^2^)

where Fr typically stands for radial force. It is one of the three main components of the cutting force and acts perpendicularly to the cutting edge, pushing the tool away from the workpiece (the other two components are the tangential force, Ft, and the axial force, Fa). Figure 3a presents an example of the cutting forces Fx, Fy, Fz, and Fr after following the above procedures, while Figure 3b shows how the original data for the cutting force are filtered and corrected in the case of the cutting force Fz.

As the Al-Li alloy is the base alloy in this study, the statistical analysis using ANOVA for this alloy will be presented first as a reference point, so that the effect of alloying additions and heat treatments applied to the subsequent Al-Li-Cu and Al-Li-Cu-Sc alloys may be brought out when applying the ANOVA technique to these two alloys.

In the statistical analysis of the cutting force data for the Al-Li base alloy, the ANOVA results presented in Table 4 reveal essential information regarding the effect of parameters such as the feed rate, cutting speed, hardness, and cooling used in the study and their interactions. Specifically, the model developed efficiently explains 96.27% of the observed variability in cutting force, indicating a clear correlation between the cutting parameters selected and the resulting cutting force.

**Figure 4:**
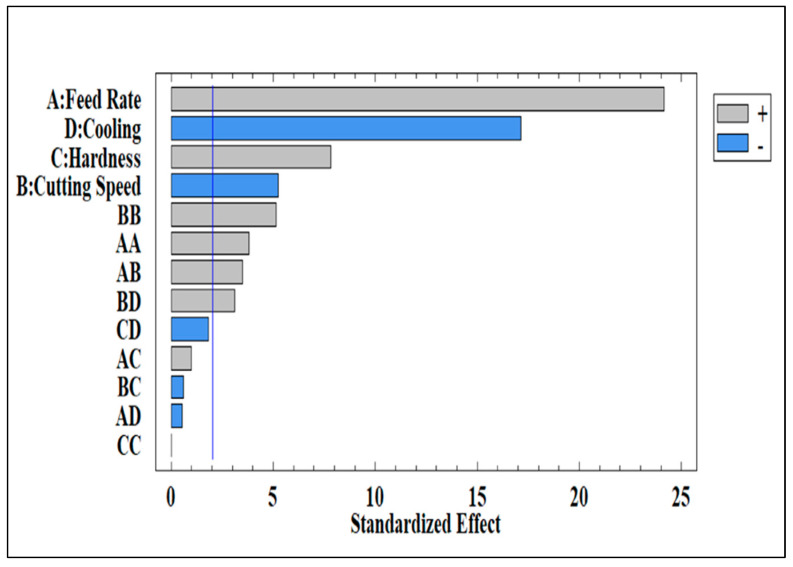
Pareto Chart of cutting force variables and their interactions for Al-Li- alloy.

The Pareto Chart is a very powerful tool for showing the relative importance of the independent variables selected in relation to their effect on the dependent variables being studied. The Pareto Chart shown in Figure 4 illustrates the independent variables used in this study and their interactions. As may be observed, all parameters are found to exceed the threshold (i.e., the 80% cut-off line) indicated by the vertical blue line.

### 3.2. Effect of Feed Rate on Cutting Force

#### 3.2.1. Al-Li Alloy

The feed rate has the greatest influence on the cutting force. This observation is in alignment with the findings of Songmene et al. [18] and Akram et al. [19], where higher feed rates resulted in greater cutting forces due to the removal of thicker chips and increased material volume.

**Figure 5:**
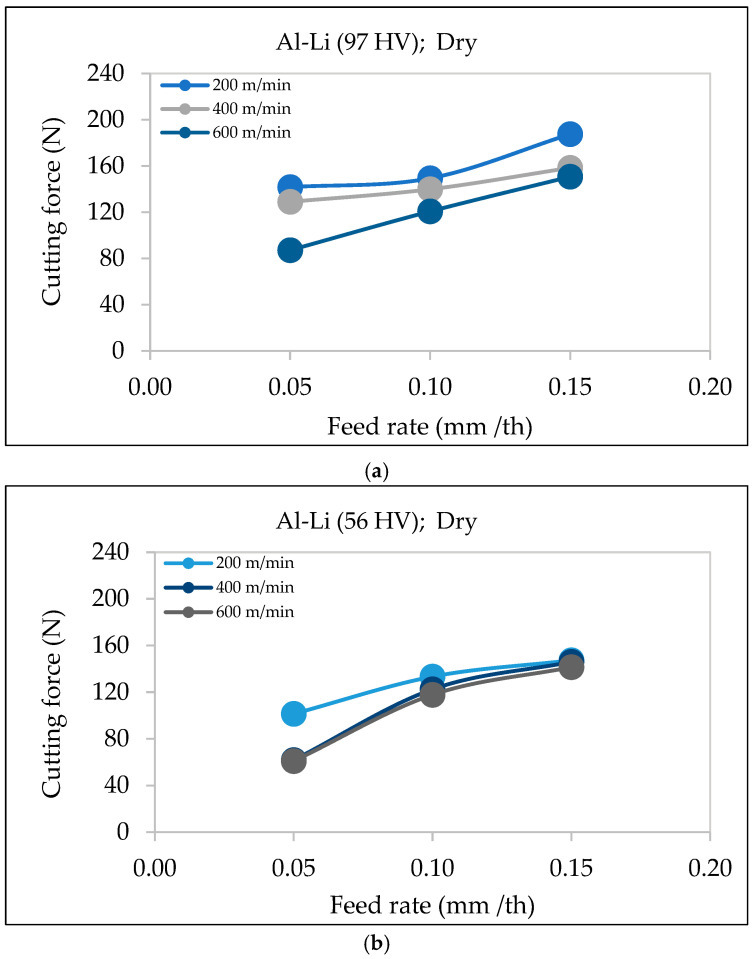
Variation in cutting force as a function of feed rate under different cutting speeds during dry machining of Al-Li alloy with (**a**) hardness ~56 HV, (**b**) hardness ~97 HV.

Figure 5 illustrates the relation between the cutting force and feed rate for Al-Li alloys with different hardness values (97 HV and 56 HV) under dry machining conditions. In both cases, an increase in feed rate leads to a rise in the cutting force, confirming the trend reported in previous studies. For the Al-Li alloy with higher hardness (97 HV), the cutting forces are generally higher compared to the alloy with lower hardness (56 HV). This may be credited to the increased resistance of the alloy with higher hardness to plastic deformation, requiring greater force to shear the material.

Additionally, at an increased feed rate, the rise in the cutting force is more pronounced at lower cutting speeds (e.g., 200 m/min) compared to higher speeds (600 m/min). This behavior is due to thermal softening effects at higher speeds, which counteract some of the force increase associated with the feed rate. Furthermore, elevated feed rates generate more heat from the increased friction between the tool and the workpiece, leading to localized softening. However, the extent of this effect varies based on the alloy hardness, as seen in the differing slopes of the cutting force curves.

#### 3.2.2. Al-Li-Cu Alloy

**Figure 6:**
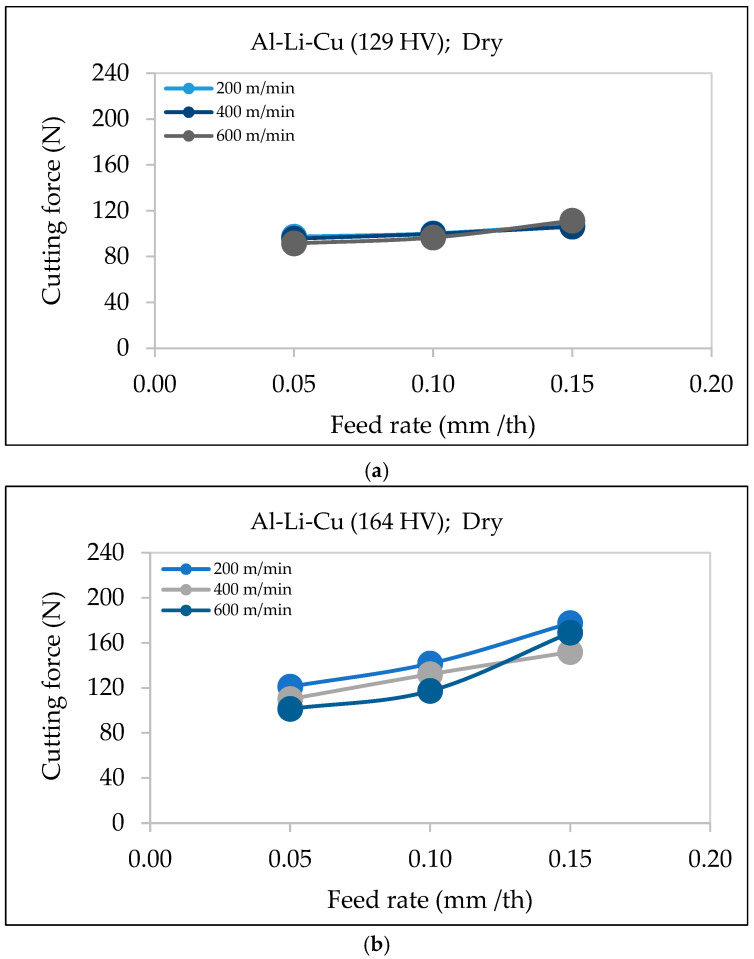
Variation in cutting force as a function of feed rate under different cutting speeds during dry machining of Al-Li-Cu alloy with (**a**) hardness ~129 HV, (**b**) hardness ~164 HV.

As the feed rate increases, the cutting force also increases. This trend is highlighted in the graphs displayed in Figure 6 for Al-Li-Cu alloys with hardness values of 129 HV and 164 HV. In both cases, increasing the feed rate leads to a noticeable rise in the cutting force.

In the Al-Li-Cu alloy with 129 HV (Figure 6a), the cutting force exhibits a moderate increase across different feed rates (0.05, 0.10, and 0.15 mm/th), although the impact is relatively mild, particularly at higher cutting speeds (600 m/min). However, in the alloy with higher hardness (164 HV), the cutting force increases more significantly, particularly at lower speeds (200 m/min, as observed from Figure 6b. This behavior indicates that materials with higher hardness require greater cutting force due to their increased resistance to material removal, consistent with findings by Kiswanto et al. [20] and Gökkaya [21].

Higher feed rates lead to greater material removal per unit time and larger chip formation, which necessitate more force to shear and remove the material. This trend is typical in machining aluminum alloys and is supported by studies by Venkatesan et al. [22] and Srivastava et al. [23].

#### 3.2.3. Al-Li-Cu-Sc Alloy

**Figure 7:**
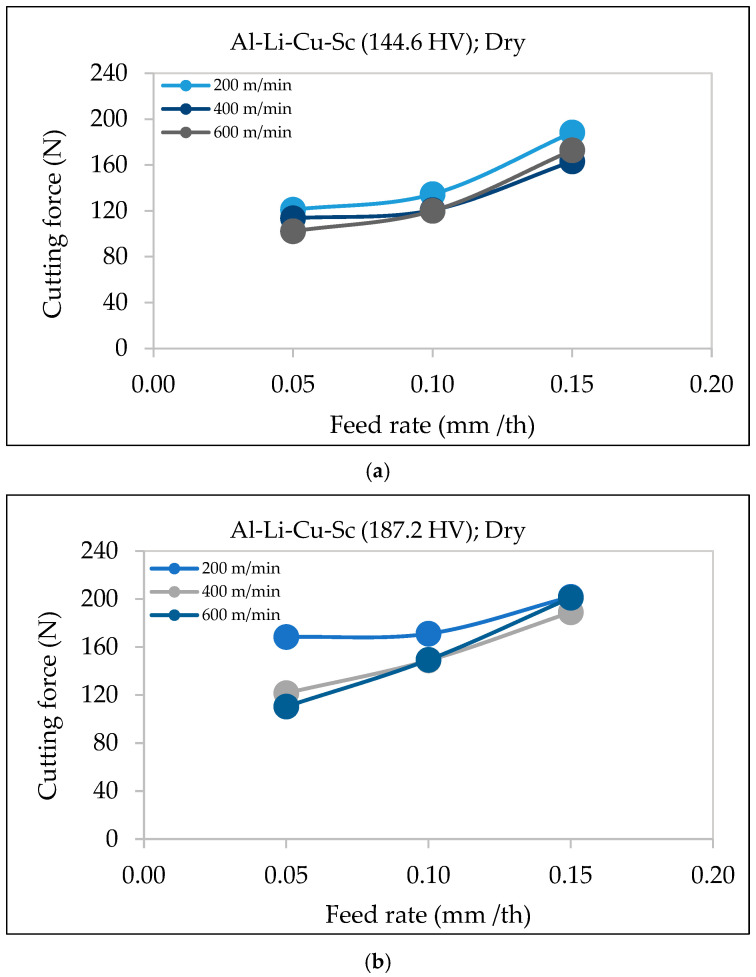
Variation in cutting force as a function of feed rate under different cutting speeds during dry machining of Al-Li-Cu-Sc alloy with (**a**) hardness ~144 HV, (**b**) hardness ~187 HV.

In the Al-Li alloy with Cu and Sc additions and a hardness of 144.6 HV (Figure 7a), the cutting force increases steadily as the feed rate rises from 0.05 to 0.15 mm/th. The effect is more pronounced at lower cutting speeds (200 m/min), where the chip load and resistance are higher. As the cutting speed increases, the rise in the cutting force becomes less significant, indicating better chip formation and reduced cutting resistance at higher speeds.

For the alloy with higher hardness (187.2 HV) shown in Figure 7b, the cutting force increases more sharply, especially at lower cutting speeds. This reflects the increased material strength, which leads to greater resistance and thicker chip formation, as observed by Zha et al. [24] and Wang et al. [25]. Similarly, Sequeira et al. [26] noted that higher feed rates increase cutting forces in aluminum alloys due to thicker chips, which require greater energy to shear.

Overall, the feed rate directly impacts the cutting force in Al-Li-Cu-Sc alloys. The plots in Figure 7 reveal that higher-hardness alloys demand greater cutting force at the same feed rates. This underscores the importance of selecting optimal feed rates to balance productivity, machining efficiency, and tool wear, particularly in high-strength aluminum–lithium alloys.

### 3.3. Effect of Hardness on Cutting Force

#### 3.3.1. Al-Li Alloy

**Figure 8:**
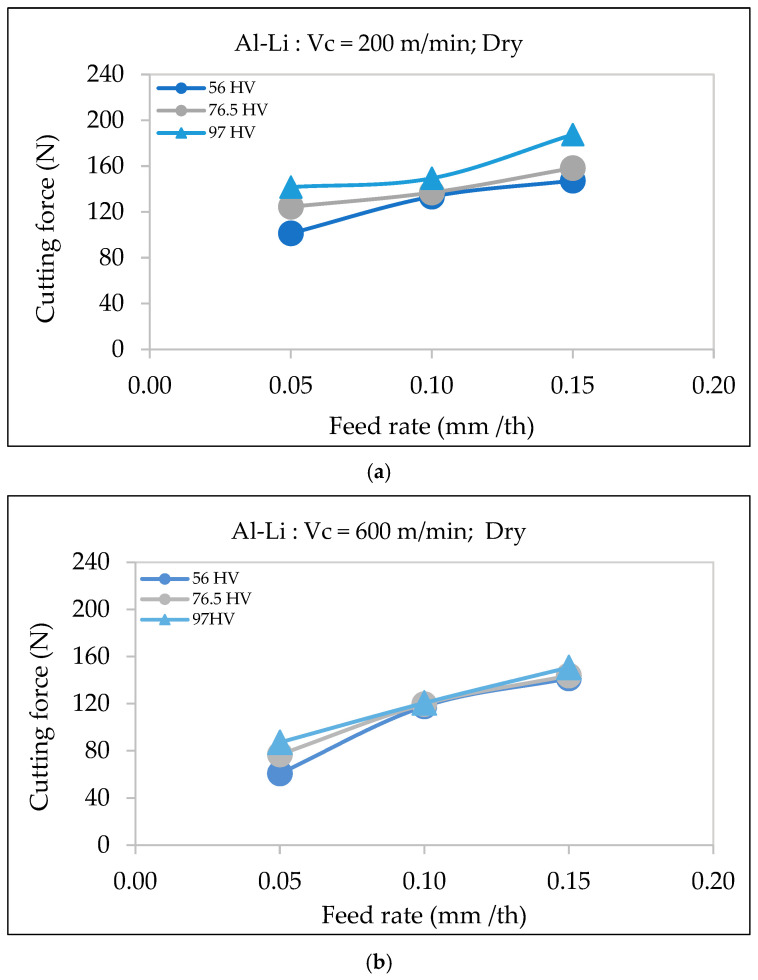
Variation in cutting force as a function of feed rate during dry machining of Al-Li alloys of different hardness: (**a**) cutting speed = 200 m/min, (**b**) cutting speed = 600 m/min.

Hardness plays a significant role in determining the cutting force, with alloys of higher hardness requiring more force during machining. For the Al-Li alloys, the cutting force increases when machining material at different hardness levels (56 HV, 76.5 HV, and 97 HV) and cutting speeds of 200 m/min and 600 m/min, as shown in Figure 8.

In the first graph displayed in Figure 8a, the cutting force increases steadily as the feed rate rises from 0.05 to 0.15 mm/th for a cutting speed of 200 m/min. The alloy with the highest hardness (97 HV) exhibits the greatest cutting force across all feed rates, while the alloy with the lowest hardness (56 HV) has the lowest cutting force. This is due to the increased resistance to deformation in harder materials, which requires more energy for chip formation, consistent with findings by Pathak et al. [21] and Gökkaya [27].

In the second graph, Figure 8b, although the overall cutting force is somewhat reduced due to the higher cutting speed (600 m/min) and improved chip evacuation, the trend remains the same: the cutting force increases with both the feed rate and the hardness. Higher-hardness materials, due to their greater strength, resist cutting, which results in thicker chips that require higher forces for shearing and material removal.

#### 3.3.2. Al-Li-Cu Alloy

**Figure 9:**
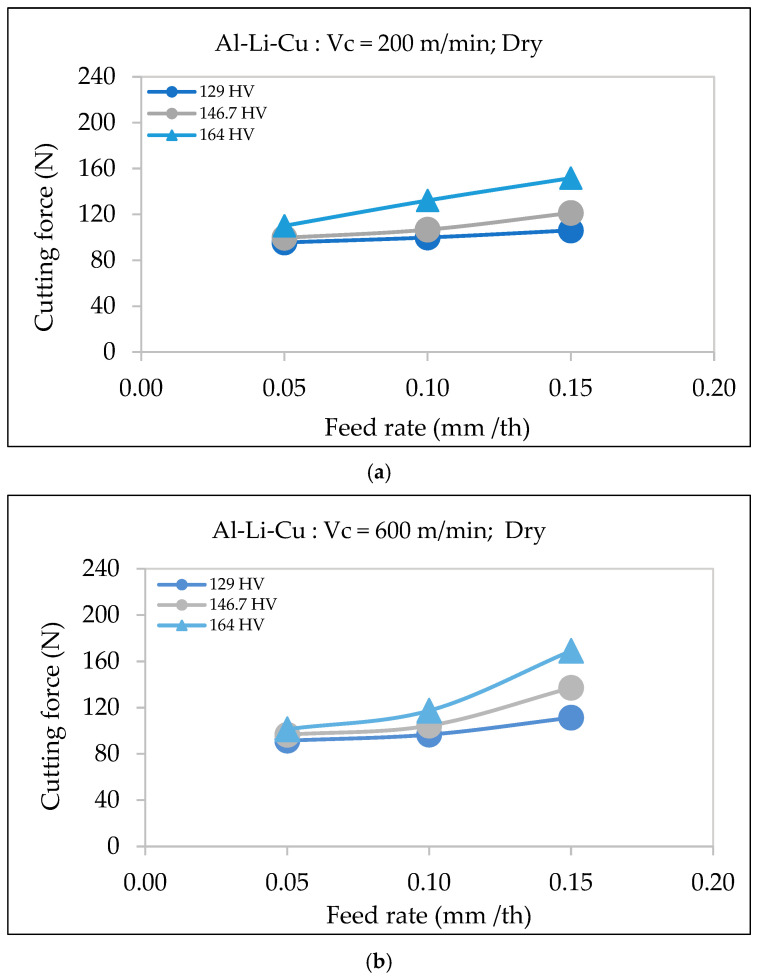
Variation in cutting force as a function of feed rate during dry machining of Al-Li-Cu alloys of different hardness: (**a**) cutting speed = 200 m/min, (**b**) cutting speed = 600 m/min.

Hardness significantly affects the cutting force, with alloys of higher hardness (164 HV) requiring greater force than those with lower hardness (129 HV). As shown in Figure 9, at a cutting speed of 200 m/min, the cutting force increases with an increase in feed rate, meaning that the Al-Li-Cu alloy with 164 HV requires the highest force across all feed rates.

The same trend is observed at the higher cutting speed of 600 m/min, where the difference in cutting force between the alloys possessing the highest and lowest hardness values become even more noticeable at higher feed rates. This indicates that the effect of hardness on cutting force is magnified at higher speeds, likely due to the increased resistance to shearing displayed by the harder material.

This correlation is supported by El-Kady et al. [28] and Kaya et al. [29], who found that increased hardness in aluminum alloys and composites raises cutting resistance due to greater material strength. In the case of Al-Li-Cu alloys, the presence of copper-rich precipitates (e.g., θ′ or T1 phases) further contributes to increased cutting forces, as these precipitates enhance alloy strength by impeding dislocation movement, making the material more resistant to shearing.

#### 3.3.3. Al-Li-Cu-Sc Alloy

**Figure 10:**
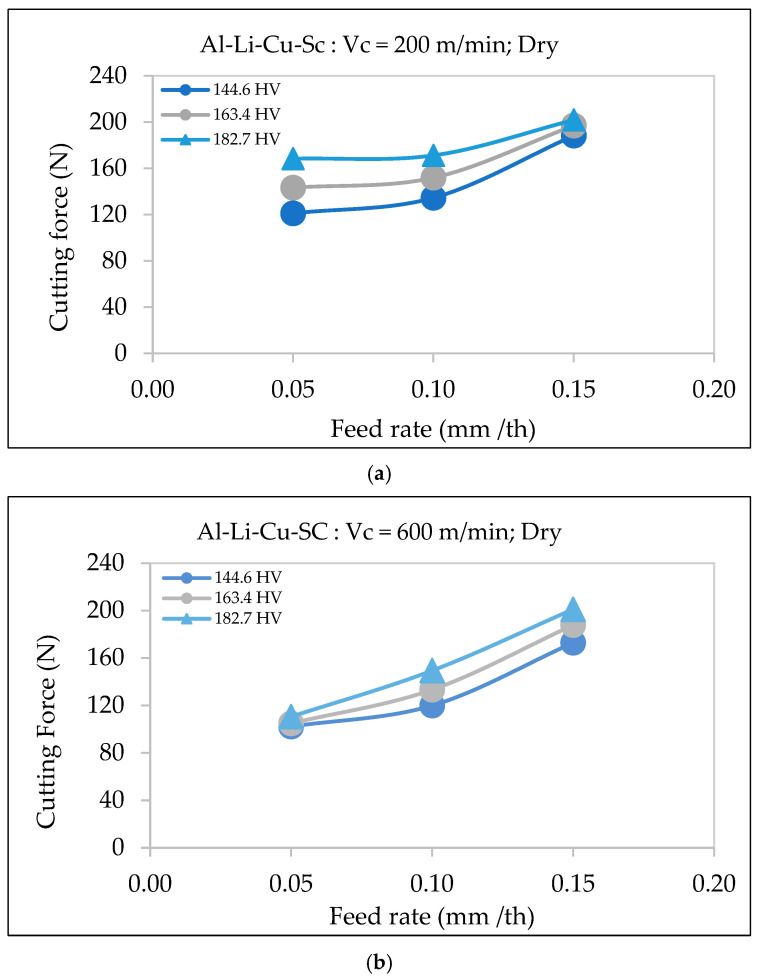
Variation in cutting force as a function of feed rate during dry machining of Al-Li-Cu-Sc alloys of different hardness: (**a**) cutting speed = 200 m/min, (**b**) cutting speed = 600 m/min.

Hardness significantly affects the cutting force, with materials of higher hardness (182.7 HV) requiring greater force than those with lower hardness (144.6 HV). As shown in Figure 10, at a cutting speed of 200 m/min, the cutting force increases with the feed rate, with the 182.7 HV alloy always requiring the highest force for every feed rates. At the higher speed of 600 m/min, the cutting force rises more sharply, especially at high feed rates, suggesting that the impact of hardness on cutting resistance becomes more pronounced at elevated speeds.

The increase in cutting force is due to the greater resistance of materials with higher hardness to deformation, as highlighted by Kaya et al. [29] and Tash et al. [30,31]. Hardness changes caused by heat treatments alter the alloy’s microstructure, increasing cutting forces. For Al-Li-Cu-Sc alloys, the presence of fine scandium-rich precipitates further enhances the hardness, making the material more resistant to shearing and increasing the cutting force required during machining.

### 3.4. Effect of Lubrication on Cutting Force

#### 3.4.1. Al-Li Alloy

**Figure 11:**
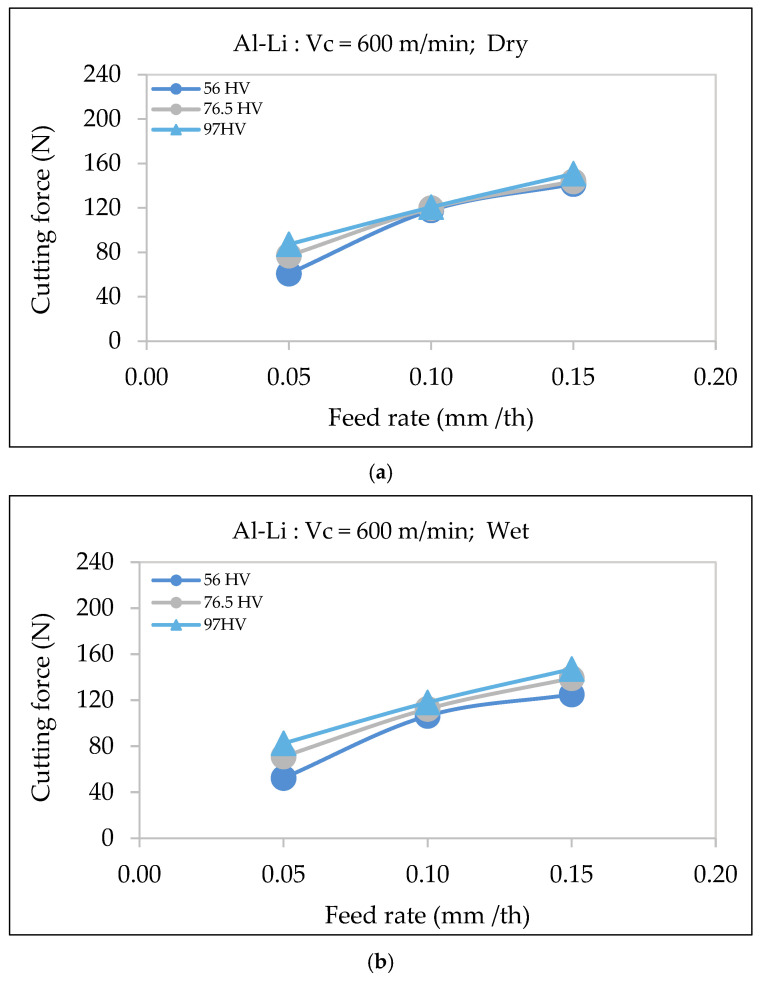
Variation in surface roughness as a function of feed rate for Al-Li alloys of different hardness using a cutting speed of 600 m/min under (**a**) dry and (**b**) wet machining conditions.

With regard to machining conditions (dry vs. wet), lubrication appears to have a negligible impact on the cutting force, as noted from Figure 11. At a cutting speed of 600 m/min, the cutting forces in both dry and wet conditions exhibit a similar trend, increasing with the feed rate. The difference between the dry and wet conditions is minimal, indicating that lubrication does not significantly reduce the cutting force for the base Al-Li alloys. This is obvious even at higher feed rates, where the cutting force curves for dry and wet conditions remain nearly parallel, with only slight variations.

This observation contrasts with studies by Kishawy et al. [32], where wet cooling reduced cutting forces by lowering the tool temperature. However, the unique properties of the Al-Li alloy, such as its lower thermal conductivity and reduced tendency toward built-up edge formation, could well contribute to its consistent cutting behavior, regardless of the cooling conditions.

#### 3.4.2. Al-Li-Cu Alloy

In Al-Li-Cu alloys, wet cooling reduces the cutting force compared to dry machining. This reduction is attributed to improved lubrication and chip evacuation, as well as stabilized thermal gradients that prevent the localized softening of the workpiece. These findings align with studies by Namlu et al. [33] and Kouam et al. [34], which demonstrated that wet machining reduced the cutting force through effective cooling and lubrication.

**Figure 12:**
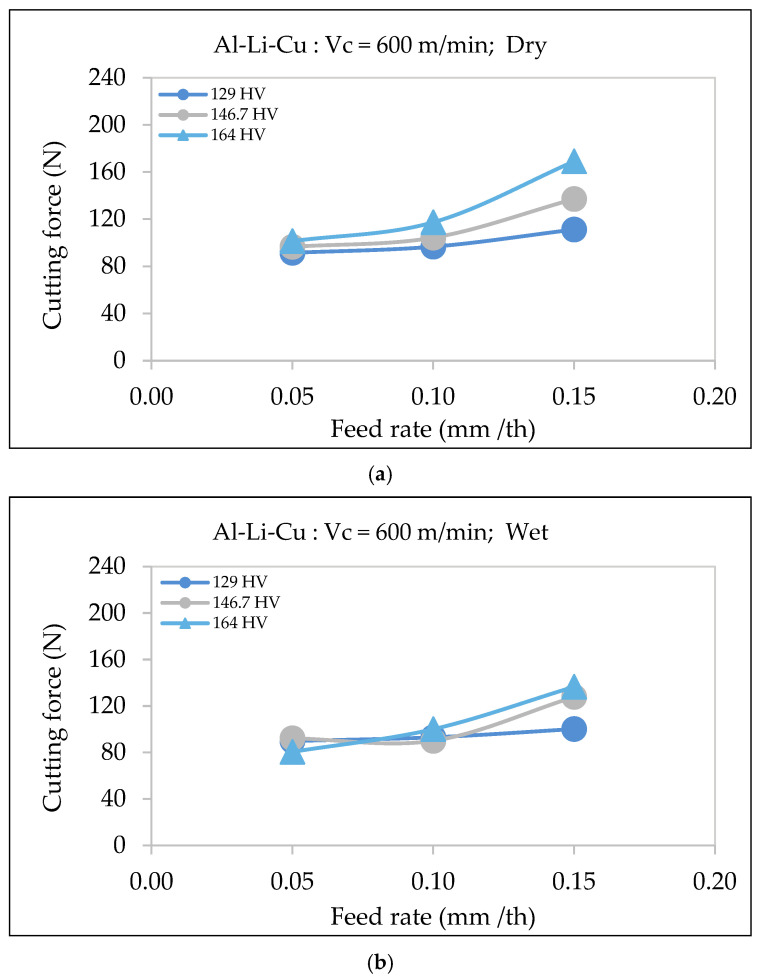
Variation in surface roughness as a function of feed rate for Al-Li-Cu alloys of different hardness, using a cutting speed of 600 m/min, under (**a**) dry and (**b**) wet machining conditions.

As shown in Figure 12, the cutting force increases with the feed rate for both dry and wet conditions. However, wet machining results in consistently lowering the cutting force for all hardness levels. The reduction in cutting force is more significant for alloys with higher hardness. For instance, in dry conditions, the cutting force for the alloy with the highest hardness (164 HV) is significantly higher than in wet conditions, where cooling mitigates the thermal degradation of precipitates, preserving alloy hardness and minimizing cutting resistance.

Moreover, the difference in cutting force between wet and dry conditions is more evident at increased feed rates, especially in alloys exhibiting higher hardness values. This suggests that cooling plays a critical role in stabilizing cutting forces in those alloys with higher hardness by preventing an excessive rise in temperature from leading to a rise in softening.

These results reaffirm the importance of lubrication and cooling in machining Al-Li-Cu alloys, particularly in applications where precision and tool longevity are critical.

#### 3.4.3. Al-Li-Cu-Sc Alloy

**Figure 13:**
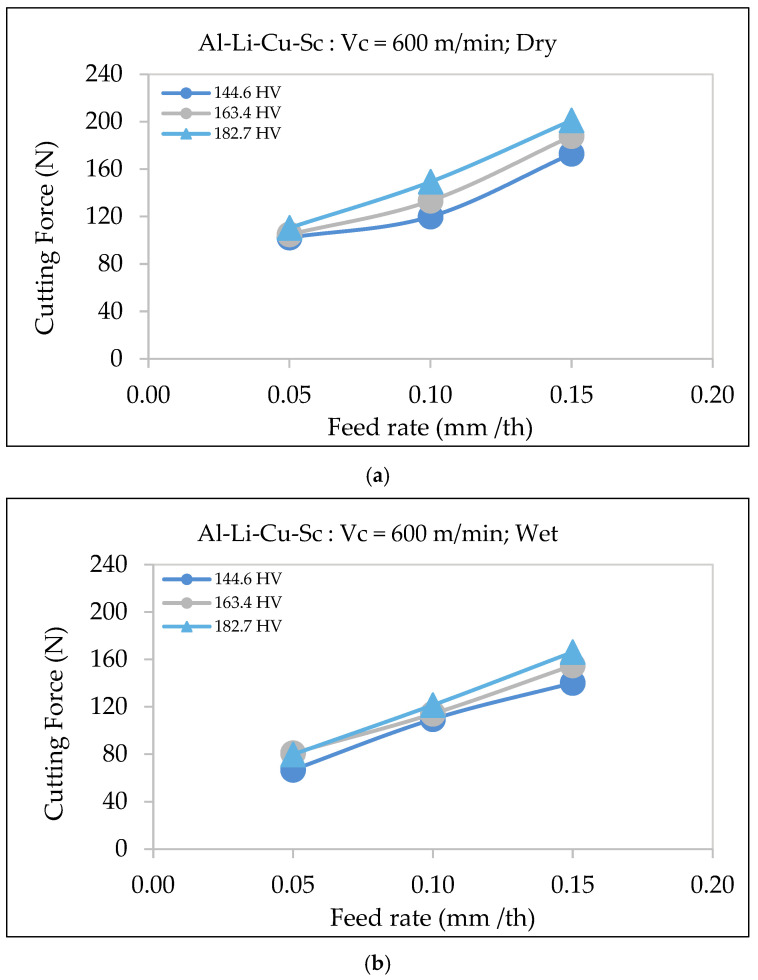
Variation in surface roughness as a function of feed rate for Al-Li-Cu-Sc alloys of different hardness, using a cutting speed of 600 m/min, under (**a**) dry and (**b**) wet machining conditions.

The use of wet machining in the case of Al-Li-Cu-Sc alloys also reduces the cutting forces considerably, as demonstrated by a comparison between the two types of machining conditions, displayed in Figure 13. Under dry conditions (Figure 13a), the cutting force increases more steeply with the feed rate and hardness, reaching values of over 200 N at a feed rate of 0.15 mm/th for the hardest alloy (182.7 HV). In contrast, wet machining consistently mitigates the cutting force across all hardness levels and feed rates.

At higher feed rates (0.15 mm/th), wet machining lowers the cutting force, as seen from Figure 13b. This reduction can be attributed to the cooling fluid’s ability to decrease friction, minimize heat generation, and reduce tool wear, aligning with previous findings by Boswell et al. [35] and Jebaraj et al. [36]. Furthermore, lubrication helps stabilize the microstructure of Al-Li-Cu-Sc alloys by preventing thermal degradation and localized softening, which is particularly crucial in alloys with higher hardness.

Figure 13b illustrates that under wet conditions, the cutting force increases more gradually with the feed rate, reducing the variability. For lower-hardness alloys (144.6 HV), the difference in cutting force between dry and wet machining conditions is less pronounced due to their lower sensitivity to thermal effects. However, for the alloys with higher hardness (163.4 HV and 182.7 HV), wet machining provides substantial benefits by maintaining material integrity and reducing cutting force variability, particularly at high feed rates.

### 3.5. Interaction Between Effective Factors in Cutting Force

#### 3.5.1. Al-Li Alloy

The feed rate and cutting speed interactions considerably affect the cutting force. At higher speeds (600 m/min), the cutting force is lower across the range of feed rates, with a notable difference of 53 N at lower feed rates (0.05 mm/th), which diminishes to 20 N at the 0.15 mm/th feed rate. Similarly, hardness and feed rate interactions show increased cutting force for materials with higher hardness, though the differences diminish slightly at higher feed rates.

**Figure 14:**
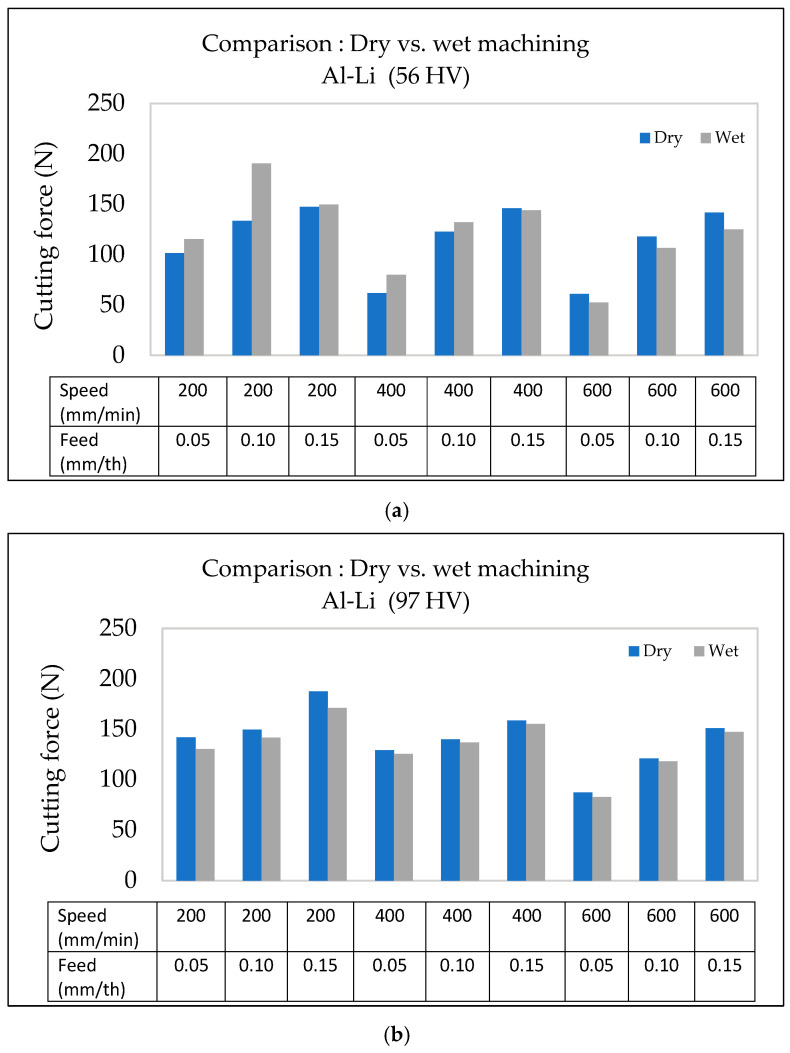
Comparison of surface roughness as a function of feed rate and cutting speed in Al-Li alloys under dry vs. wet machining conditions: (**a**) 56 HV, (**b**) 97 HV.

As illustrated in Figure 14, wet machining consistently results in lower cutting forces compared to dry machining, particularly at lower speeds and feed rates. However, as the speed and feed rate increase, the difference between dry and wet conditions narrows. This suggests that the effect of lubrication is more pronounced at lower cutting parameters, where friction and heat generation are higher in dry conditions.

For the softer Al-Li alloy (56 HV), the cutting force exhibits a significant variation with the feed rate under dry conditions, peaking at 0.10 mm/th and then slightly decreasing at 0.15 mm/th. This trend is less pronounced with wet machining, where cooling stabilizes the cutting force needed. In contrast, for the harder Al-Li alloy (97 HV), the cutting force remains relatively stable across all feed rates, although at higher levels than in the alloy with lower hardness, reaffirming the role of hardness in influencing cutting resistance.

**Figure 15:**
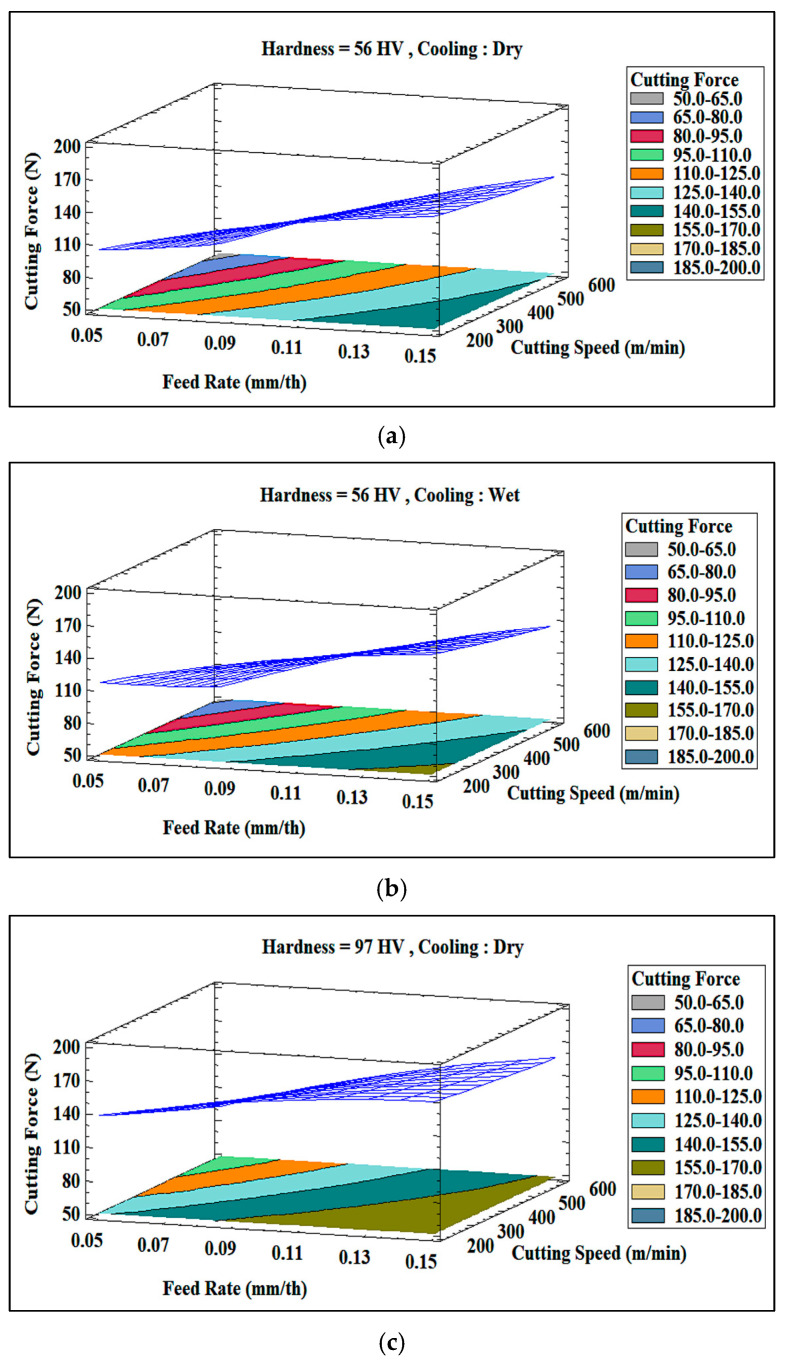

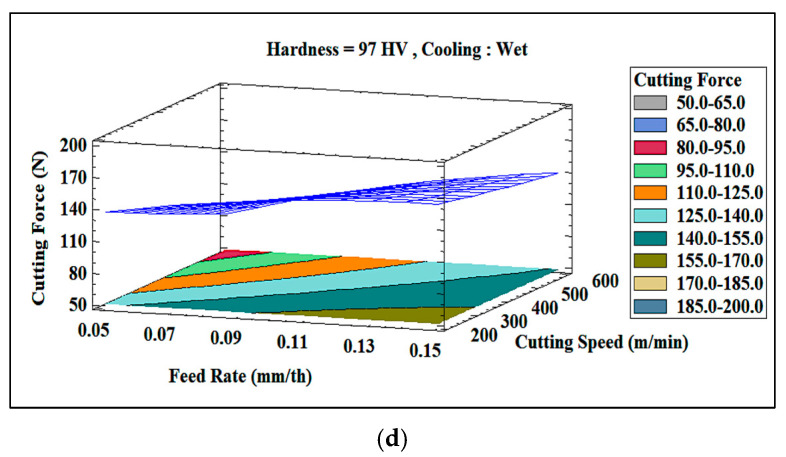
Three-dimensional surface plots of cutting force Rt obtained for Al-Li alloys exhibiting hardness values of (**a**,**b**) 56 HV, (**c**,**d**) 97 HV under (**a**,**c**) dry, and (**b**,**d**) wet machining conditions.

Transitioning from dry to wet conditions results in a relatively small reduction in cutting force, as noted from Figure 15a,b. This trend persists even at higher alloy hardness levels, as demonstrated by Figure 15c,d. However, with an increase in hardness, a slight but noticeable change occurs, which is more pronounced in dry conditions and at lower feed rates (0.05 mm/th) and higher cutting speeds (600 m/min). These findings are in agreement with a previous study by Boswell et al. [35], confirming that cooling primarily enhances process stability rather than drastically reducing the cutting force.

#### 3.5.2. Al-Li-Cu Alloy

**Figure 16:**
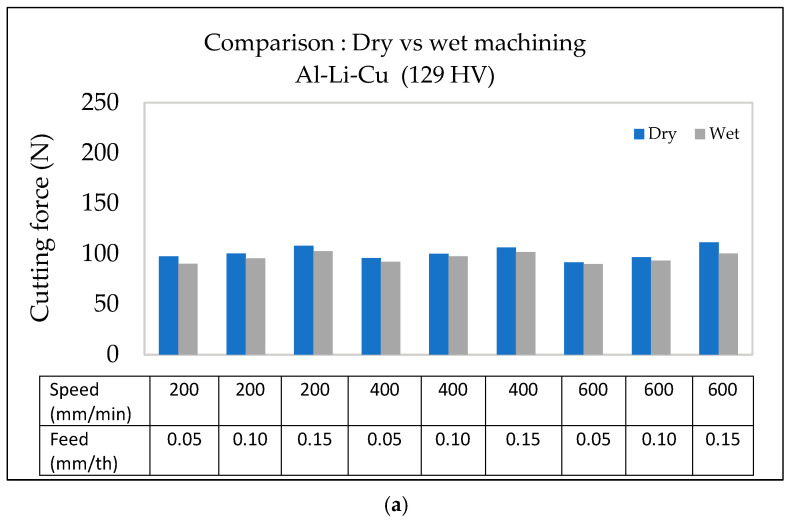

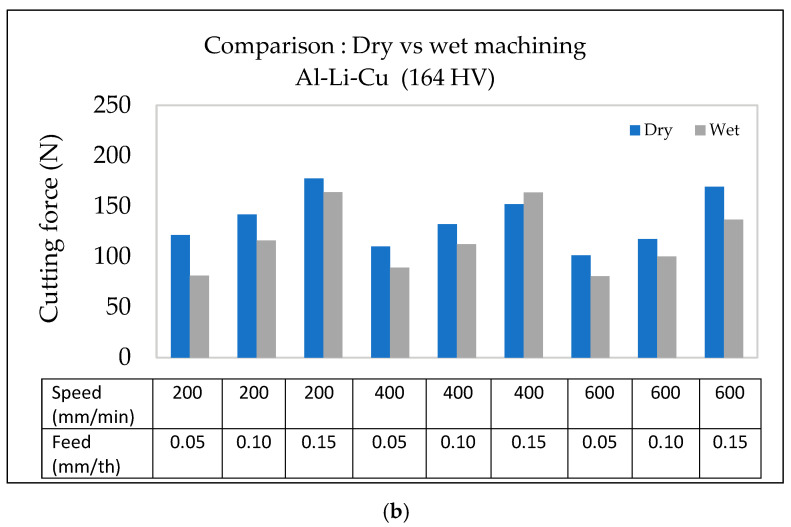
Comparison of surface roughness as a function of feed rate and cutting speed in Al-Li-Cu alloys under dry vs. wet machining conditions: (**a**) 129 HV, (**b**) 164 HV.

The interaction between key machining parameters (feed rate, alloy hardness, and machining environment) significantly affects the cutting force when machining Al-Li-Cu alloys. Figure 16a,b clarify how these factors vary between alloys of different hardnesses (129 HV and 164 HV) under the two machining environments.

At a low feed rate of 0.05 mm/th, and for both alloy hardness levels, the cutting forces obtained are comparable, at around 95 N. However, as the feed rate is increased to 0.15 mm/th, the harder Al-Li-Cu alloy (164 HV) requires a much higher cutting force, exceeding that for the softer alloy (129 HV) by over 60 N. This can be attributed to the greater resistance to deformation in the alloy with higher hardness, exacerbated by the increased chip load at higher feed rates.

The cooling mode (machining environment) also plays a crucial role in mitigating cutting forces, especially for the alloy with higher hardness. For the alloy with lower hardness (129 HV), the difference in cutting force in the two cases remains minimal, as the microstructure is less prone to thermal degradation. Conversely, for the alloy with higher hardness (164 HV), wet machining reduces the cutting force by approximately 20 N. This indicates that wet cooling stabilizes the microstructure under high thermal loads, maintaining consistent hardness and reducing localized thermal softening.

**Figure 17:**
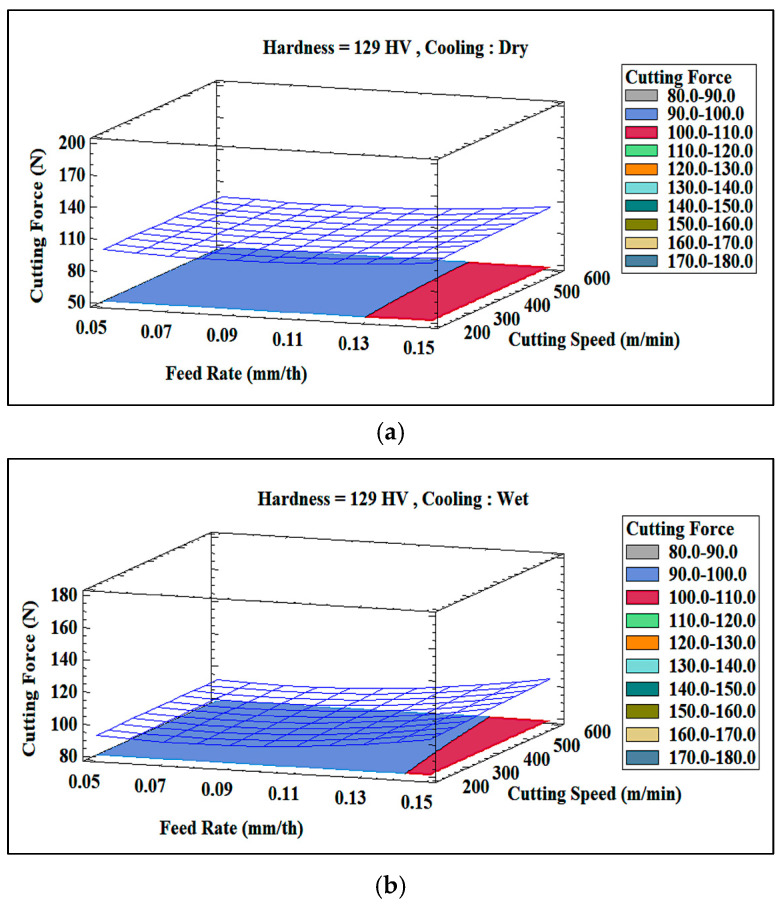

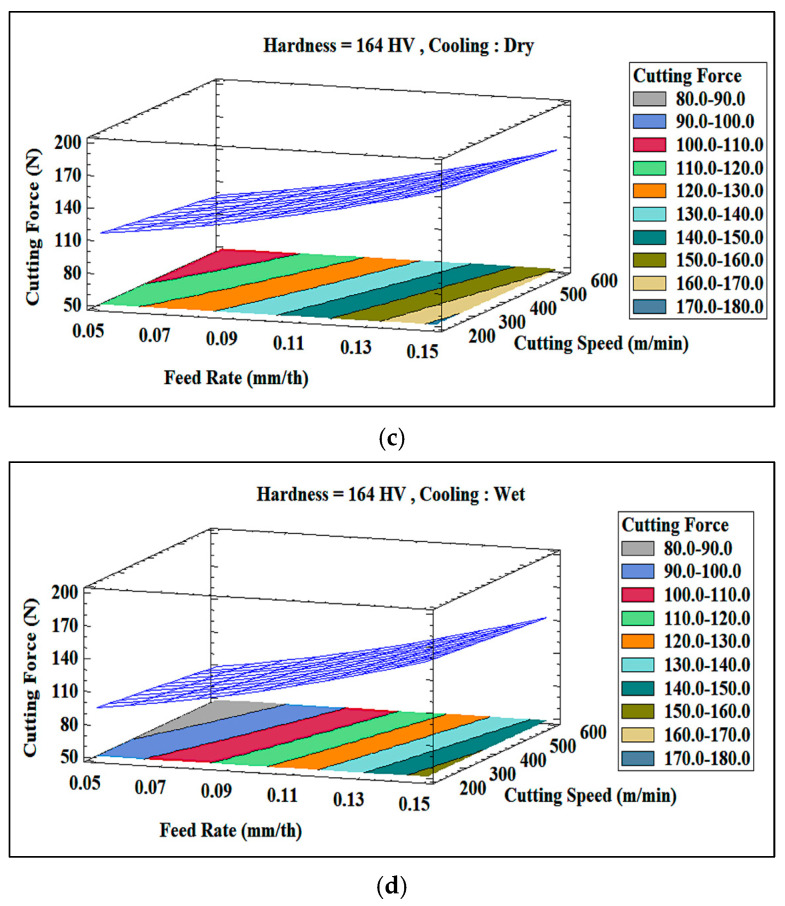
Three-dimensional surface plots of cutting force Rt obtained for Al-Li-Cu alloys exhibiting hardness values of (**a**,**b**) 129 HV, (**c**,**d**) 164 HV under (**a**,**c**) dry, and (**b**,**d**) wet machining conditions.

The 3D plots in Figure 17 further demonstrate that the cutting force increases more sharply with the hardness at higher feed rates. Wet machining notably dampens this effect for the harder (164 HV) alloy, illustrating the role of the cooling fluid in temperature regulation. For the softer (129 HV) alloy, however, the cooling impact remains relatively negligible due to the lower sensitivity of the alloy to thermal variations.

#### 3.5.3. Al-Li-Cu-Sc Alloy

**Figure 18:**
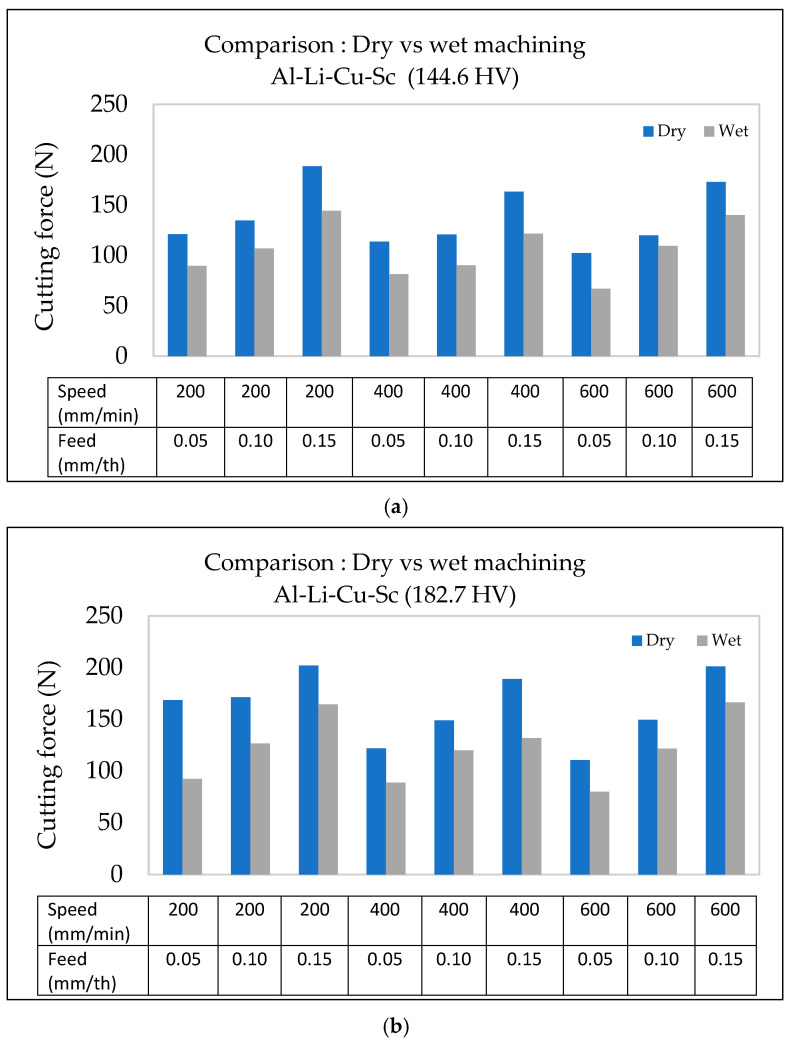
Comparison of surface roughness as a function of feed rate and cutting speed in Al-Li-Cu-Sc alloy under dry vs. wet machining conditions: (**a**) 144.6 HV, (**b**) 182.7 HV.

Figure 18 demonstrates how the cutting forces are impacted significantly by the interactions among the cutting parameters of feed rate and cutting speed, the alloy hardness, and the cooling mode during machining in the case of Al-Li-Cu-Sc alloys. Figure 18a,b show these interactions for alloys with hardness levels of 144.6 HV and 182.7 HV, respectively.

At low feed rates (0.05 mm/th), the cutting force is reduced by approximately 25 N upon increasing the cutting speed from 200 mm/min to 600 mm/min. This reduction is attributed to the thinner chips generated at higher speeds, which decrease the material resistance. Additionally, wet machining consistently diminishes the cutting force at all cutting speeds by improving lubrication and reducing heat buildup, as seen in Figure 18.

**Figure 19:**
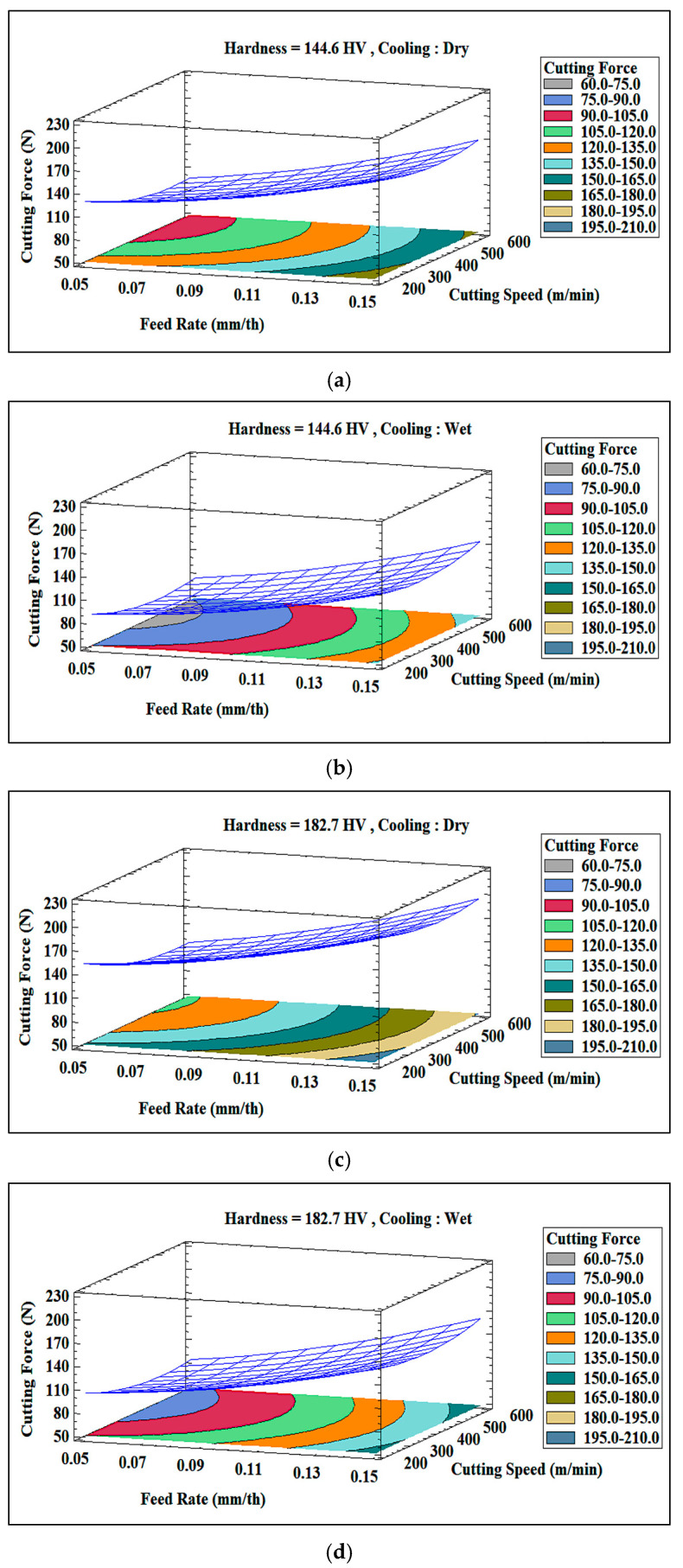
Three-dimensional surface plots of cutting force Rt obtained for Al-Li-Cu-Sc alloys exhibiting hardness values of (**a**,**b**) 144.6 HV, (**c**,**d**) 182.7 HV under (**a**,**c**) dry, and (**b**,**d**) wet machining conditions.

The 3D plots in Figure 19 show that the combined effect of hardness and feed rate is more pronounced at higher feed rates (0.15 mm/th), where the alloy with higher hardness (182.7 HV) experiences significantly higher cutting forces than the alloy with lower hardness (144.6 HV). Under dry machining conditions, thermal effects increase resistance, resulting in a sharper rise in cutting force for the alloy with higher hardness (see Figure 19a,c). However, wet machining reduces this disparity by stabilizing the microstructure and minimizing localized thermal softening, particularly at lower feed rates (see Figure 19b,d).

Figure 19 also confirms that both increased hardness and feed rate substantially elevate the cutting force, especially in dry machining. Wet machining (cooling) moderates these effects, keeping the cutting forces relatively constant for the softer alloy and significantly reducing the increase for the harder alloy, particularly under low feed rates, where thermal effects are less dominant.

## 4. Conclusions

The work reported in this article concentrated on the machining of Al-Li and Al-Li-based alloys containing Cu and Cu and Sc additions to determine how machining variables such as the feed rate, cutting speed, machining environment (lubrication), and alloy material hardness would affect the cutting force behavior. Tests were conducted on 162 machined samples and analyzed to elucidate how these parameters would influence cutting forces during the milling process. Based on the findings from cutting force measurements, a comprehensive evaluation was performed to understand the interactions between the machining conditions and the mechanical properties of the alloys.

1. At the highest hardness (182.7 HV), the Al-Li-Cu-Sc alloy requires a cutting force of 128.45 N, which is lower than the corresponding values determined for the Al-Li-Cu alloy (164 HV, needing a cutting force that is 27.66 N higher) and the Al-Li alloy (97 HV, needing a cutting force that is 19.15 N higher). At its lowest hardness (144.6 HV), the Al-Li-Cu-Sc alloy also outperforms the other two alloys, requiring a cutting force of only 108.15 N. These values were obtained at 600 m/min cutting speed, and a feed rate of 0.15 mm/th, under wet machining conditions.
2. The interaction between hardness and feed rate significantly influences the cutting force in the Al-Li-Cu-Sc alloy, making it more sensitive to hardness changes. The interaction between feed rate, cutting speed, hardness, and cooling mode significantly impacts the cutting force during the machining of Al-Li-Cu-Sc alloys.
3. The Al-Li-Cu-Sc alloy shows a notable reduction in cutting force with the increase in cutting speed, a trend less pronounced in the base and Cu-containing Al-Li alloys. High cutting speeds (600 m/min) and low feed rates (0.05 mm/th) consistently result in lower cutting forces and better surface quality for all alloys, with the Al-Li-Cu-Sc alloy benefiting the most.
4. Wet machining significantly reduces the cutting force, with the Al-Li-Cu-Sc alloy showing the greatest improvements. Cooling (lubrication) stabilizes microstructural properties, particularly in the presence of scandium, reducing tool wear and ensuring predictable machining outcomes.
5. The scandium in the Al-Li-Cu-Sc alloy refines the grain structure and forms stable precipitates (e.g., δ′ and T1), improving the hardness and mechanical properties while maintaining excellent machinability.
6. The statistical model developed for the present alloys explains the variability in cutting force successfully (96.27%), ensuring reliable and predictable machining performance. Optimal machining conditions, including high cutting speeds and wet machining, provide the best results for all alloys, with Al-Li-Cu-Sc benefiting the most.

Overall, the Al-Li-Cu-Sc alloy outperformed the others, requiring lower cutting forces due to its refined grain structure and stable precipitates enhanced by the addition of scandium. It showed consistent performance across different hardness levels and benefited most from high cutting speeds and wet machining, which reduced the cutting forces and improved the machining predictability. These findings highlight the superior machinability of these alloys and their suitability for aerospace applications.

## Figures and Tables

**Table 1:** Chemical compositions of the Al-Li alloys studied [16].

Element (wt%) *													
Alloy	Si	Mg	Cr	Mn	Fe	Cu	Zn	Ni	Ti	V	Li *	Zr	Sc
Al-Li	0.58	<0.003	<0.003	0.027	0.28	0.23	0.040	0.010	0.052	0.012	1.9	0.3	<0.010
Al-Li-Cu	0.18	<0.003	<0.003	0.012	0.099	2.9	0.039	0.007	0.090	0.010	3.0	0.3	<0.010
Al-Li-Cu-Sc	0.17	<0.003	<0.003	0.011	0.10	2.6	0.039	0.007	0.067	0.010	2.9	0.3	0.185

* Analyzed by OES.

**Table 2:** Details of heat treatments employed for the Al-Li-based alloys [16].

Alloy	Solution Heat Treatment Temp (°C)/Time (h)	Aging Temp. (°C)	Aging Time (h)
Al-Li	580 °C/1 h	130, 150	1, 2.5, 5, 10, 15, 25, 35, 45
Al-Li-Cu	505 °C/5 h	160, 180, 200	5, 10, 15, 20, 25, 30
Al-Li-Cu-Sc	505 °C/5 h	160, 180, 200	5, 10, 15, 20, 25, 30

**Table 3:** Experimental variables and their levels for the three Al-Li alloy systems studied.

Variable	Al-Li Alloy	Al-Li-Cu Alloy	Al-Li-Cu-Sc Alloy
Cutting Speed (m/min)	200, 400 and 600	200, 400 and 600	200, 400 and 600
Feed Rate (mm/th)	0.05, 0.10 and 0.15	0.05, 0.10 and 0.15	0.05, 0.10 and 0.15
Hardness (HV)	56	129	144.6
	76.5	146.7	163.4
	97	164	182.7
Cooling Mode	Dry, Wet		
Depth of Cut (mm)	2		

**Table 4:** Table showing the Al-Li base alloy ANOVA for cutting force.

Source	Sum of Squares	Df	Mean Square	F-Ratio	*p*-Value
A: Feed Rate	35,218.8	1	35,218.8	582.65	0.00
B: Cutting Speed	1670.08	1	1670.08	27.63	0.00
C: Hardness	3708.81	1	3708.81	61.36	0.00
D: Cooling	17,759.8	1	17,759.8	293.81	0.00
AA	881.79	1	881.79	14.59	0.00
AB	742.59	1	742.59	12.29	0.00
AC	57.97	1	57.97	0.96	0.33
AD	16.27	1	16.27	0.27	0.61
BB	1593.14	1	1593.14	26.36	0.00
BC	21.85	1	21.85	0.36	0.55
BD	588.87	1	588.87	9.74	0.00
CC	0.04	1	0.037	0.00	0.98
CD	200.69	1	200.69	3.32	0.08
Total error	2417.84	40	60.45		
Total (corr.)	64,878.5	53			

## Data Availability

The raw data supporting the conclusions of this article will be made available by the authors on request.
